# Coping with stigma: the experiences of Chinese patients living with lung cancer

**DOI:** 10.1186/s40064-016-3486-5

**Published:** 2016-10-13

**Authors:** Huaxia Liu, Qianqian Yang, Georgia L. Narsavage, Chunling Yang, Yue Chen, Guiying Xu, Xia Wu

**Affiliations:** 1 School of Nursing, Taishan Medical University, Tai’an, Shandong China; 2 University of South Carolina College of Nursing, Columbia, SC USA; 3 Nursing Department of Liaocheng People’s Hospital, Liaocheng, Shandong China; 4 Oncology Department of Liaocheng People’s Hospital, Liaocheng, Shandong China

## Abstract

**Purpose/objectives:**

To describe the experiences of stigma and coping strategies among patients with lung cancer in China.

**Research approach:**

Qualitative.

**Setting:**

The oncology department at Liaocheng Peoples Hospital.

**Participants:**

A purposive sample of 17 patients experiencing stigma related to lung cancer voluntarily participated in data collection.

**Methodologic approach:**

Individual, semistructured qualitative interviews were chosen. Participants completed about a 30-min focused interview. Exploratory qualitative approach guided data analysis.

**Findings:**

Three main thematic elements emerged from the interview data:(1) sources of stigma, such as smoking, decreased ability to work, difficulties caring for self and family, damage to self-image, and cough and expectoration; (2) experiences of stigma, including feelings of stigma, remorse, loss of dignity, uselessness, social isolation, perceived exclusion, rejection, and discrimination; and (3) coping strategies, such as concealing the fact of sickness, reducing social activities, seeking medical assistance, adhering to treatment, and disclosing dissatisfaction.

**Conclusions:**

Our results indicate the presence of perceived stigma among patients with lung cancer. Future work should address the stigma associated with lung cancer and its related factors.

**Interpretation:**

As point-of-care providers, staff nurses are well positioned to develop effective interventions to help patients deal with stigma and to accomplish the goal of providing holistic nursing care.

## Background

Lung cancer is the most frequent cause of cancer death among men and women worldwide (Juan et al. 2015; Wille et al. 2016). In 2008 in China, lung cancer replaced liver cancer as the leading cause of death among cancer patients and the Chinese lung cancer registry reported that lung cancer accounted for 22.7 % of all malignancies (She et al. 2013).

Advances in research along with newer treatment protocols have increased life expectancy (Huang et al. 1993). With increased life expectancy, patients and their families are having to cope with the challenges of living with a disease that is often perceived by others, and even the patients themselves, to have been caused by a “bad” behavior–smoking. While recognizing the deadly role that smoking causes in lung cancer development, emphasizing an individual’s smoking history (or non-smoking history)underpins blame and is not effective in reducing or coping with the disease’s associated mark of disgrace or “stigma” of smoking (Chambers et al. 2012). As smoking has become more socially unacceptable there has been a significant increase in negative mood among smokers, who are experiencing shame, blame, and stigma (Stuber et al. 2009). Stigma itself may be “stacked” when the negative event (having lung cancer) is linked with the stigma of smoking, with increased rejection, exclusion, alienation, or discrimination by others as a result of a disease and its perceived cause (Liu and Wang 2012). Lung cancer patients experience the perception of being blamed for smoking as the “stigma”, in a very different context than personally believing smoking caused their lung cancer. A growing body of evidence suggests that lung cancer stigma (LCS) not only affects the psychological health, but also the physical health of lung cancer patients (Chapple et al. 2004; Cataldo et al. 2012).

In 2014, A Chinese version of the Cataldo Lung Cancer Stigma Scale (CLCSS-CV) was used to assess the stigma experienced by Chinese lung cancer patients, and researchers found that “lung cancer patients in China generally experience some form of social stigma as a result of their disease” (Yang et al. 2014). Nonetheless, the nature and essence of the Chinese patient’s experience of coping with the “stigma” of lung cancer is not known. The purpose of this research was to describe the experience of Chinese patients in coping with the stigma of a lung cancer diagnosis. A qualitative approach was used to examine the subjective experience of Chinese patient’s coping with the stigma of a diagnosis of lung cancer. We were interested in the patient’s perception of the cause of their lung cancer and how others reacted to them. The research question guiding the study was “What is the lived experience of coping with the stigma of lung cancer in China?”

In recent years, stigma related to lung cancer has become a growing area of study for researchers (Hamann et al. 2014; LoConte et al. 2008; Chambers et al. 2012). In 2004, Chapple and colleagues interviewed 45 patients with lung cancer and asked about their experiences following diagnosis (Chapple et al. 2004). Their qualitative study indicated that patients with lung cancer experienced stigma because the disease is directly attributable to the intensity of tobacco use. According to LoConte and colleagues (LoConte et al. 2008), and Lebel and colleagues (Lebel et al. 2013), lung cancer patients had higher levels of perceived cancer-related stigma than patients with prostate, head, neck, or breast cancers. Conlon and colleagues, in a study of oncology social workers’ perceptions of lung cancer experience, reported that lung cancer patients have high rates of “stacked” stigma with respect to cigarette smoking (Conlon et al. 2010). The negative emotional phenomenon of stigma increases the burden on patients. In a study of 95 patients with stage II–IV non-small-cell lung cancer, Gonzalez and Jacobsen reported that perceived stigma and depression were positively correlated (r = 0.46, P < 0.001) (Gonzalez and Jacobsen 2012). Cataldo and colleagues also reported the presence of an inverse relationship between LCS and quality of life (r = 0.65, P < 0.001) (Cataldo et al. 2011). To better measure perceived stigma and its effects on patients with lung cancer, Cataldo and colleagues developed and evaluated the Cataldo Lung Cancer Stigma Scale (CLCSS) (Cataldo et al. 2011), a reliable and valid measure of health-related stigma for lung cancer patients. Each stigma item was measured using a four-point Likert-type scale ranging from 1 (strongly disagree) to 4 (strongly agree). Ratings are summed to form a cumulative score. The 46 item CLCSS scale potential range of scores was 46–184 with a higher score meaning more stigma (Cataldo et al. 2012). A Chinese version of the CLCSS scale (CLCSS-CV) has been used to identify stigma in China. The CLCSS-CV includes 27 items and 4 subscales. Each item was measured through a four-point Likert scale ranging from 1 (strongly disagree) to 4 (strongly agree), and two items were scored inversely. Higher scores indicate a stronger stigma perceived by patients. For the Chinese version, the score was the mean of all items answered (sum/# items answered). The average score on the CLCSS-CV obtained in testing was (2.68 ± 0.40). As tested by Yang and colleagues, the values for the CLCSS-CV from 2.29 to 2.68 indicate a low level of stigma; 2.69 to 3.08 indicate moderate stigma, and a score of >3.09 indicates a high level of stigma (Yang et al. 2014). To purposely obtain a sample of patients for the current study, 2.29 as a cut-off score was used to identify patients who experienced “stigma”.

Although the measurement of perceived stigma has been well-studied using quantitative methodology (LoConte et al. 2008; Chambers et al. 2012; Yang et al. 2014; Hamann et al. 2014), quantitative research by its very nature is constrained to studying a limited number of variables and may not capture the full experience of living with a disease. A related and independent phenomenon involves coping with the stigma of lung cancer, including changes in identities, roles, family bonds, and patterns of behavior. Routine treatment of lung cancer is rigorous, complicated, and exhausting. Dealing with complex radiation and chemotherapy regimens as well as surgery in some cases can prove to be very stressful for patients and their family members. This stress is compounded when the perceived stigma of having the disease results in isolation from others and disrupts daily life and social interactions (Yang et al. 2014). The patient’s lived experience of coping with stigma may be in areas not captured in quantitative research. There are few qualitative research studies on living with lung cancer that address the experience of living with the stigma of this extremely complex multivariate disease, and no studies were identified for the Chinese population.

In summary, there is a body of quantitative research documenting that stigma is experienced by lung cancer patients, including those living in China. Understanding the experience of Chinese patients’ coping with the stigma has not been studied. Qualitative research approaches are best suited to describing the phenomenon of coping with the stigma of lung cancer. Helping nurses to understand how patients live with the disease and cope with related stigma may change practice.

## Methods

### Participants and procedure

The study was an exploratory qualitative analysis using guided interviews. Although no USA formal Institutional Review Board was available, Taishan Medical University and Liaocheng Peoples’ hospital ethics committee approved the study (#201306).

Participants were recruited based on their first-hand experience with the phenomenon of interest (stigma) to allow for descriptions of the life experiences of coping with the stigma of lung cancer. Patients were eligible to take part in the study if they were age 20 years or older, diagnosed with lung cancer by pathological examination, aware of their diagnosis, identified stigma (at least 2.29: possible range 1–4) on the modified Chinese version of the Cataldo Lung Cancer Stigma Scale (CLCSS-CV) (Cataldo et al. 2011; Yang et al. 2014), were awake and alert, were able to express their own opinions, native standard Chinese speakers, and they or their family members could provide informed consent.

A purposive sample of 17 patients experiencing stigma related to lung cancer voluntarily participated in data collection. Permission to do the study was also obtained from the hospital administrators and departments. Written informed consent was obtained from 6 patients and for the other 11 patients who could not complete written consent, an oral consent from the patient and a family member was obtained. Ten (10) men and seven (7) women being treated in a tertiary hospital in Shandong Province in China were recruited for this study. They had a mean age of 58.3 years (range 29–80 years). Four of the participants (23.5 %) had completed elementary school, four (23.5 %) had completed junior high school, and nine (52.9 %) had completed senior or secondary high school. Three patients with stage II cancer, seven patients with stage III cancer and seven patients with stage IV participated. More details of the participant demographics are shown in Table 1.

**Table 1:** General demographic information for the 17 participants

Participant no.	Sex	Age (years)	Marital status	Geographic location	Education (years)	Cancer stage	Treatment	Length of lung cancer diagnosis (months)
1	Male	65	Married	Rural	Junior high school	III	Surgery, chemotherapy	6
2	Male	56	Married	Urban	Secondary school	III	Surgery, radiotherapy	18
3	Female	66	Married	Rural	Elementary school	IV	Surgery	12
4	Female	53	Married	Urban	Secondary school	IV	Chemotherapy	15
5	Male	49	Divorce	Urban	Senior high school	III	Surgery, chemotherapy	5
6	Male	70	Married	Urban	Secondary school	IV	Chemotherapy	9
7	Female	80	Married	Urban	Junior high school	IV	Traditional Chinese medicine	24
8	Male	68	Married	Rural	Junior high school	III	Surgery	14
9	Male	43	Married	Urban	Secondary school	II	Chemotherapy	5
10	Male	68	Married	Rural	Elementary school	IV	Chemotherapy	9
11	Male	29	Married	Urban	Senior high school	II	Surgery, chemotherapy	5
12	Female	72	Married	Urban	Senior high school	IV	Surgery, radiotherapy	14
13	Male	65	Married	Urban	Junior high school	II	Surgery, chemotherapy	6
14	Female	48	Married	Rural	Elementary school	III	Chemotherapy	11
15	Female	54	Married	Rural	Elementary school	IV	Surgery, radiotherapy	8
16	Male	69	Married	Urban	Senior high school	III	Chemotherapy	13
17	Female	36	Married	Urban	Senior high school	III	Surgery, chemotherapy	8

### Data generation and analysis process

The researcher and assistants had administered the CLCSS-CV to lung cancer patients and assisted patients having difficulty reading to complete the scale as part of a previous study (Yang et al. 2014). If “stigma” was identified, and other inclusion criteria were met, after obtaining consent of the patients and family members present in the hospital setting as needed for oral consent, focused interviews were conducted with the patients.

The first author did all the interviews assisted by the head nurse of the tumor departments at Liaocheng Peoples Hospital. An interview guide (see Table 2) consisting of open-ended questions was used. Questions for the interview were generated in advance by the researchers and, although not piloted with patients, were reviewed by expert nurses familiar with the problem of cancer stigma to support content validity. The guide was used to elicit personal descriptions of the experience of coping with stigma in living with lung cancer. Sensitivity to issues of vulnerability and physical limitations for patients with lung cancer and their families required modification of data collection methods–exhaustive interviews were not possible. Although six questions were generated in advance, due to the breathlessness associated with the patient’s conditions, after the first interview, the interview guide was shortened to three questions (#3, #5, and # 6) that were used for “focused” 30 min interviews and any of the remaining three questions were only used if time and the patient’s condition allowed. Interviews were conducted at the bedside, audio recorded and transcribed. Detailed field notes were kept as well. Thus adapting the qualitative process to the patient’s real-world limitation in breathing, we were able to capture the experience in the patient’s own words-staying true to the intent of qualitative research. The investigators met to read the transcripts and discuss emerging themes. The interview questions were as follows: BOLD questions (#3, #5, and #6) are the three asked first and thus were most often used.

**Table 2:** Selected questions used in guided data collection

Focus questions
(1) What has it been like for you since you were diagnosed with lung cancer? What were the attitudes of others immediately following your diagnosis?
(2) When you experience negative emotions, how do you adjust? What factors have helped you to overcome the psychological distress?
*(3) Have you encountered unfair treatment in your daily life since you were diagnosed with lung cancer? Please provide an example*
(4) How do you feel when you suffer from discrimination, dislike, rejection, and alienation? Please use a few examples to illustrate your feelings
*(5) How did your feelings about the stigma you have experienced affect your work, study, and life?*
*(6) When suffering from discrimination, dislike, rejection, and alienation, how do you respond?*

The data analysis process involved multiple steps. The interview transcripts were verified for accuracy and completeness by comparing them to the recordings, and then read repeatedly by the first author and discussed and reviewed with the other authors. Significant comments related to the patient’s experiences of stigma and coping with stigma were extracted, followed by description of the meanings. Identified themes then emerged from the aggregated meanings. Tables 3, 4 and 5 provide examples of significant statements that were responses to the questions, organized by the corresponding themes and sub-themes. Original words from the transcripts were examined to confirm each theme. The researchers then assimilated results, and formulated the description of experience of stigma and coping with stigma for Chinese patients hospitalized with lung cancer.

**Table 3:** Sources of stigma

Themes	Findings and supporting quotes
1.1 Smoking	Cigarette smoking represents a primary risk factor for lung cancer. More than 80 % of diagnoses occur in current or former smokers. The participants often expressed regret at having smoked
	Participant 2: “*I started smoking at age 16. So far, I have smoked for 40* *years. Now I have the disease and it feels that I am being punished! I have spent a lot of money in treating the disease, I feel very guilty. I think that if I had not smoked at that time, it would have been better*”
	Participant 3: “*I have smoked for 40* *years, and now I have the disease. It’s a punishment. If only I had not smoked at the beginning. I feel very guilty and much remorse.*” Although approximately 80–90 % of all lung cancer cases are smoking related, some patients do not have a history of smoking (Gómez et al. 2007). However, participants reported that people around them believed that these patients had lung cancer because of smoking
	Participant 11: “*A few good friends came to see me last week. They told me lung cancer is related to smoking. They act as though it is my fault that I have lung cancer. However, I have never smoked. I felt quite upset*”
1.2 Decreased ability to work	Due to lung cancer, patients may find they have reduced physical abilities, meaning they are unable to work as hard as they did previously
	Participant 15: *“The company had planned to give me a promotion to general manager. The board of directors heard from someone that I got lung cancer, so my boss didn’t promote me. Ah, that’s because of the disease…”*
1.3 Difficulties caring for self and family	The participants reported being very weak after receiving treatment for their disease and needing care from their families after hospital discharge. Not only were they unable to care for themselves, but they were also unable to care for their families as they had previously. As a result, the participants reported developing feelings of shame and remorse
	Participant 1: *“It doesn’t matter how I have suffered. I am afraid that the disease will cause problems for my grandson. I haven’t been able to take care of my grandson”*
	Participant 8: *“Now I cannot afford any help for my children. Instead, I trouble them a lot…I felt even guiltier”*
	Participant 9*: “I cannot even help my children”*
1.4 Damage to self-image	Lung cancer itself and chemotherapy for treatment with related side-effects can cause hair loss, weakness, and disfigurement, leading to a negative self-image. These changes were identified as a cause of stigma
	Participant 3: *“After my first chemotherapy treatment, a lot of hair fell out. When I was out shopping, others asked me ‘What’s wrong with your hair?’ I felt they whispered about me even when I didn’t notice them talking”*
	Participant 4: *“My hair has fallen out; my legs were probably invaded by metastatic disease. Now I walk with a limp, not a good image”*
	Participant 9: *“Now my hair has fallen out and I always vomit because of chemotherapy. Now I have a poor quality of life and I have no dignity”*
	Participant 14: *“I have a bad cough. As soon as I cough, my family will frown. So I usually try to cough as little as I can possibly help”*
	Participant 17: *“The cancer cells spread to the breast, so the side of the breast was removed; I feel so ashamed”*

**Table 4:** Experiences of stigma

Themes	Findings and supporting quotes
1.1 Perceived discrimination	In the interviews, the patients talked about their experiences of being rejected and discriminated against by their families, friends, other patients, colleagues, supervisors and neighbors
	Participant 1: *“When the cleaner gave back my patient robes, she wore gloves and a face mask, and washed her hands with soap several times. This is discriminating against and detesting me, isn’t it?”*
	Participant 2: *“Because my kids are studying abroad, the company arranged for a colleague to take care of me. But I feel he is afraid of me and always tries to avoid me. Once I saw him secretly asking the head nurse whether the disease was infectious”*
	Participant 10: *“The patient sharing the room with me had gastric carcinoma. I was coughing. He thought my disease was contagious and tried to avoid me. Last time, his granddaughter came to see him. He did not let her enter the room. Later, I underwent chemotherapy with the side effects of nausea and vomiting. He felt uncomfortable and asked to be transferred to another room”*
1.2 Social isolation and exclusion	In the process of interacting with others, some of the participants reported feeling excluded, often limiting their social activities
	Participant 1: *“I won’t go there next time if they treat me badly. During the chemotherapy a lot of hair has fallen out and I don’t feel well. I hardly go out”*
	Participant 11: *“I feel I am isolated from the rest of the world”*
	Participant 13: *“I feel I’m not as good as others because I have lung cancer. Sometimes I feel they exclude me”*

**Table 5:** Coping strategies

Themes	Findings and supporting quotes
Concealing the fact of sickness	Patients with lung cancer can suffer exclusion, rejection, and discrimination in their daily lives. In order to prevent such phenomena, patients reported concealing the fact of their sickness
	Participant 8: *“Some people mark me as ‘the source of infection’ after learning that I have lung cancer, which brings me a lot of trouble. So I know that I can’t tell others about my disease, and I ask my children not to tell others”*
	Participant 9: *“I do not talk about my disease to others easily. If my superiors are aware of my disease, can I be promoted? I wish I could arrange work for my children before I retire. I undergo chemotherapy early in the morning and after chemotherapy I go back to work as quickly as possible, trying my best to hide the secret”*
Seeking explanations to give to others	During the interviews, several participants suggested that the main reason for alienation is that others are worried that the disease can be infectious. These participants reported studying the disease in detail by reading books and consulting doctors and nurses. They then explain the condition to others, and tell them that lung cancer is not contagious
	Participant 2: *“I repeated several times that the disease was not contagious. Don’t be afraid”*
Being cooperative patients	Because the source of LCS is related to a perception that patients themselves are responsible, those who want to reduce the stigma may try to be good, “cooperative” patients, following the treatment plan and actively caring for themselves, in hopes of an early hospital discharge and resumption of social and family roles
	Participant 6: *“I want to cure the disease quickly and leave the hospital soon”*
	Participant 9: *“I cooperate with the doctors and nurses actively. I hope to get out of the hospital soon, so that I cause my children less trouble and reduce my feeling of guilt”*
Disclosing dissatisfaction	Patients can feel discontent and anger if their treatment results are not as they hoped, if they had high expectations of recovery, or if they feel rejection and alienation from the outside world. These patients may not cooperate with treatment
	Participant 10: *“During that time I had a bad temper; I broke the thermos and cup, refused to receive nursing care, and was always angry with the medical staff”*

The rigor of the study (credibility/validity and dependability/reliability) was supported through confirmability and transferability (Cope 2014). Confirmability was achieved through the use of an audit trail and review of raw data by the other researchers. Transferability refers to the probability that the study findings have meaning to others in similar situations (Miyata and Kai 2006). The comparison of current findings to previous research is included.

### Findings

Three themes of stigma and coping strategies were derived from the experiences of the participants: (1) sources of stigma; (2) experiences of stigma; and (3) coping strategies.

These findings are presented below.

#### Theme 1: sources of stigma

Many patients experience stigma due to smoking, decreased ability to work, difficulties caring for self and family, damage to self-image. An elaboration of these themes with supporting quotes can be found in Table 3.

#### Theme 2: experiences of stigma

Two kinds of experiences of stigma are perceived discrimination, and social isolation and exclusion. An elaboration of these themes with supporting quotes can be found in Table 4.

#### Theme 3: coping strategies

Study participants experiencing stigma primarily used four kinds of coping strategies including concealing the fact of sickness, seeking explanations to give to others, being cooperative patients, and disclosing dissatisfaction. An elaboration of these themes with supporting quotes can be found in Table 5.

## Discussion

### Sources of stigma

By organizing and analyzing the interview materials, the following four sources of Lung Cancer Stigma (LCS) were derived from the experiences of the participants: smoking, decreased ability to work, difficulties caring for self and family, and damage to self-image.

*Smoking* increases the risk of lung cancer and is one of the most specific risk factors identified so far. Therefore, regardless of whether an individual’s particular lung cancer is caused by smoking, most people will believe that smoking is the cause. Cognitive psychology suggests that this leads to others having a poor impression of lung cancer patients. In addition, the participants in this study reported feeling extremely regretful, angry, or inferior as a result of ignoring their own health, refusing to give up smoking, or not paying attention to their health for a long period before developing lung cancer. These feelings often co-existed, causing a large amount of psychological interference and leading to decreased will to live, which is consistent with the findings of Bell and colleagues (Bell et al. 2010).

In terms of *decreased ability to work*, although the participants reported receiving care from leaders and colleagues in the workplace, they felt that it was difficult to deliver on serious commitments, or that leaders would not delegate important, complicated, and particularly long-term work to them, resulting in a negative impact on their careers. This stigma of not being able to do their job can delay or prevent their return to work for a long time after their illness is treated.

In terms of the *difficulties caring for self and family*, many patients with lung cancer are middle-aged and are the mainstay of the family when healthy, supporting the elderly, raising children, and supplying money for family spending. With a lung cancer diagnosis, middle-aged patients can lose their ability to supply money for the family, and may become unable to take care of their children. Additionally, other family members have to spend a great deal of time caring for the patient. In line with this, patients might feel guilt toward their parents, spouses, and children, especially when accepting care from their families is perceived negatively along with limited resources due to paying high medical expenses. Feelings of shame can mean that some people are disappointed in themselves, which might even lead to despair and suicide. There have been a reports in recent years indicating that some patients with cancer commit suicide, based on the belief that, although they will not be able to continue to support their family, their death will provide some release from exhaustion and constraint for relatives, and that the family’s assets will be preserved (Villano et al. 2015).

As far as *damage to self*-*image* is concerned, chemotherapy and other lung cancer treatments can lead to hair loss and a deterioration in general appearance and manner, resulting in a sense of loss and self-abasement. Many patients with lung cancer have a cough and sputum production resulting in not only physical and psychological pain for patients, but also in feelings of inferiority. Patients reported feeling that they’re “unclean” and they worry that they might be further loathed, excluded, alienated, or discriminated against by others. Debilitation following treatment often results in patients needing help with eating and going to the toilet. Many patients, especially those who value neatness and propriety, will thus feel a tremendous stigma with poor-self-image. Social isolation may result from avoidance and fear. The psychological pressure of lung cancer stigma can inhibit rebuilding confidence and maintaining a good social mentality following treatment.

### Experiences of stigma

Patients with lung cancer can experience perceived discrimination, social isolation and exclusion from their colleagues, superiors, and even from friends. These experiences may be based on ignorance of cancer itself such as concerns that the disease is contagious. Additionally, people associate cancer with death. Patients with lung cancer can thus be labeled as “a source of infection” and “death”. At the same time, patients perceive discrimination and rejection with resulting negative emotional reactions. The participants in the current study used expressions such as guilt, self-accusation, shame, anxiety, and self-abasement to describe their experiences. These negative emotional experiences can have long-term consequences for patients, impairing their self-confidence, lowering their self-esteem, and leading to pessimism about the future (Chambers et al. 2012).

### Coping strategies

According to the theory of stress response (Lazarus 1993), we can divide LCS coping strategies into three categories. Stigma, as a negative psychological experience, involves three categories of coping responses: changing perceptions (emotion-focused coping) or changing behaviors (problem-focused coping), or a combination (mixed-focus coping). In order to avoid being labeled as a source of infection, or being rejected by others, patients with lung cancer may adopt mixed coping strategies to protect themselves. Link and colleagues (Link et al. 1989) historical work described how labeling can lead to negative outcomes and described several mixed-coping strategies that patients may adopt: (1) keeping the disease secret; (2) limiting participation in social activities; (3) trying to educate others; and (4) taking a stand against prejudice and discrimination. The participants in the current study documented coping strategies similar to those suggested by Link et al. (1989), thus continuing to support this historical perspective. Current study participants reduced emotional stress and psychological pressures by *concealing* the fact of sickness and limiting social activities, *seeking* information to educate others, and disclosing *dissatisfaction with discrimination*. Additionally they used strategies to reconstruct their self-image by being “cooperative” and adhering to treatment. These mixed-focus coping styles allowed them to change (cope with) their experience of stigma. Nevertheless, concealing the fact of sickness and reducing social activities can have conflicting effects. While these coping strategies may decrease or eliminate avoidance or discrimination from others, there are problems. In order to conceal the condition, the patient has to receive treatment and care in secret, which interrupts their daily routine and can interfere with receiving timely treatment. In addition, a patient who conceals the fact of sickness may not receive help from others. As a result, they may not obtain the rest necessary for coping with their illness, and can also face major psychological pressures and challenges. These patients can, in the long term, feel more lonely, afraid, and anxious than if they had disclosed their illness; consequently, they may experience increased helplessness and hopelessness (Chapple et al. 2004).

### Suggestions for clinical nursing

#### To strengthen education of the public

Prejudice and discrimination from the public against patients with lung cancer result from a lack of knowledge about the disease and misunderstanding of multiple causes of lung cancer. People may tend to believe that the disease is contagious and a sign of death. Medical staff should initiate public information campaigns by implementing lung cancer education programs in communities and schools. For example, information can be found online at http://www.lung.org/assets/documents/research/addressing-the-stigma-of-lung-cancer.pdf and http://www.lungcancercanada.ca/Lung-Cancer/Stigma.aspx. Nursing staff could also use mass media such as television, radio, and the internet; print media such as books and leaflets; and audiovisual tools such as films, and other media to provide accurate and factual information specifically about lung cancer to reduce the stigma. Such measures will help the public to acquire knowledge, correct misunderstandings about lung cancer, and improve the social environment for patients with lung cancer.

#### Reduce feelings of stigma by using cognitive therapy

Cognitive therapy is effective in treating anxiety, depression, and self-denial (Li 2011). Medical staff can apply cognitive therapy to help patients with lung cancer to identify and modify negative knowledge and beliefs, develop a positive attitude toward the disease, and gradually adjust to normal life. Nursing staff should first actively guide and educate patients to acknowledge and accept themselves. Next, nursing staff can help patients with lung cancer to enhance their self-confidence and self-esteem by identifying their inner strengths and potential in combating the disease, as well as positive events and experiences in their lives. Nurses can help patients to develop interpersonal skills and adapt ability, improved communication skills, and build resilience and an approach to disclose dissatisfaction in dealing with stigma.

#### Enhance social support

Lack of social support as a negative impact on the prognosis of disease, and serious effects on physical and mental health. Medical staff should help patients to establish effective social support networks, build a positive interpersonal communication environment, and improve their degree of social support. Communicating with patients can be an effective measure to reduce the experience of stigma (Li 2011). By supportive communication with patients, and awareness of their psychological state, feelings of fear and discrimination can be decreased. This can also help patients return to their families and workplaces, and be part of their community. Many resources can be found and used by nurses to educate and advocate for patients. For example, https://www.iaslc.org/patient-resources/advocacy-partners is a website that could be provided to government agencies, social groups, and community agencies to help the public develop a positive and caring attitude toward patients with lung cancer. It remains critical to help people to quit smoking, however, clinical and educational approaches should be offered with care so as not to increase the stigma experienced by patients with lung cancer.

### Limitations

This study has a number of limitations. The age range of the participants is 29–80 years, which is atypical for lung cancer. A younger individual with lung cancer may experience stigma very differently than an older individual and this is an atypical wide range in age of the sample. This may limit the generalizability of our results. Second, all patients who participated in the research were recruited from the same hospital in China. It might be that lung cancer patients who come from other treatment centers have different experiences. Third, the data were collected with participants in hospitals; there is a need for longitudinal research into whether a lung cancer patient’s experiences will change over longer periods of time after hospital discharge, as well as studies of “coping with stigma” with lung cancer patients in the outpatient setting.

### Implications for nursing and health policy

As point**-**of**-**care providers, staff nurses are well positioned to develop effective interventions to help patients deal with stigma and to accomplish the goal of providing holistic care.

## Conclusions

Results from this qualitative study indicate that patients with lung cancer in China experience multiple sources of stigma, and have experiences that can be categories as discrimination, social isolation and exclusion. This study adds to our understanding of the coping strategies that patients with lung cancer use to deal with stigma. As point-of-care providers, staff nurses are well positioned to develop effective interventions to help patients deal with stigma and to accomplish the goal of providing non-prejudicial nursing care.

## References

[CR1] Bell K, Salmon A, Bowers M, Bell J, McCullough L (2010). Smoking, stigma and tobacco ‘denormalization’: further reflections on the use of stigma as a public health tool. Soc Sci Med.

[CR2] Cataldo JK, Slaughter R, Jahan TM, Pongquan VL, Hwang WJ (2011). Measuring stigma in people with lung cancer: psychometric testing of the Cataldo Lung Cancer Stigma Scale. Oncol Nurs Forum.

[CR3] Cataldo JK, Jahan TM, Pongquan VL (2012). Lung cancer stigma, depression, and quality of life among ever and never smokers. Eur J Oncol Nurs.

[CR4] Chambers SK, Dunn J, Occhipinti S, Hughes S, Baade P, O’Connell DL (2012). A systematic review of the impact of stigma and nihilism on lung cancer outcomes. BMC Cancer.

[CR5] Chapple A, Ziebland S, McPherson A (2004). Stigma, shame, and blame experienced by patients with lung cancer: qualitative study. BMJ Br Med J.

[CR6] Conlon A, Gilbert D, Jones B, Aldredge P (2010). Stacked stigma: oncology social workers’ perceptions of the lung cancer experience. J Psychosoc Oncol.

[CR7] Cope DG (2014). Methods and meanings: credibility and trustworthiness of Queryqualitative research. Oncol Nurs Forum.

[CR8] Gómez RC, De Castro CJ, González BM (2007). Causes of lung cancer: smoking, environmental tobacco smoke exposure, occupational and environmental exposures and genetic predisposition. Med Clin (Barc).

[CR9] Gonzalez BD, Jacobsen PB (2012). Depression in lung cancer patients: the role of perceived stigma. Psychooncology.

[CR10] Hamann HA, Ostrof JS, Marks EG, Gerber DE, Schiller JH, Lee SJC (2014). Stigma among patients with lung cancer: a patient-reported measurement model. Psychooncology.

[CR11] Huang LR, Straubinge RM, Kahl SB, Koo MS, Alletto JJ, Fiel RJ (1993). Boronated metalloporphyrins: a novel approach to the diagnosis and treatment of Cancer using contrast-enhanced MR imaging and neutron capture therapy. J Magn Reson Imaging.

[CR12] Juan Y, Jian Z, Yong-Hui Z, Yong-Sheng C, Lu-Lu D, Thomas WK, Jian-Guo C (2015). Lung cancer in a rural area of China: rapid rise in incidence and poor improvement in survival. Asian Pac J Cancer Prev.

[CR13] Lazarus S (1993). Coping theory and research: past, present, and future. Psychosom Med.

[CR14] Lebel S, Castonguay M, Mackness G, Irish J, Bezjak A, Devins GM (2013). The psychosocial impact of stigma in people with head and neck or lung cancer. Psychooncology.

[CR15] Li J (2011) A study of stigma of people with schizophrenia and relationships with self-esteem and social support. Dissertation, Taishan Medical University, Tai’an (in Chinese)

[CR16] Link BG, Cullen FT, Struening E, Shrout PE (1989). A modified labeling theory approach to mental disorders: an empirical assessment. Am Sociol Rev.

[CR17] Liu Y, Wang Y (2012). Qualitative research on stigma feelings of families of first episode psychiatric patients. J Nurs Admin.

[CR18] LoConte NK, Else-Quest NM, Eickhoff J, Hyde J, Schiller JH (2008). Assessment of guilt and shame in patients with non-small-cell lung cancer compared with patients with breast and prostate cancer. Clin Lung Cancer.

[CR19] Miyata H, Kai I (2006). Reconsidering evaluation criteria regarding health care research: toward an integrative framework of quantitative and qualitative criteria. Nihon Koshu Eisei Zasshi.

[CR21] She J, Yang P, Hong Q, Bai C (2013) Lung cancer in China: challenges and interventions. Chest 143:1117–1126. http://www.journal.publications.chestnet.org 10.1378/chest.11-2948 23546484

[CR22] Stuber J, Galea S, Link BG (2009). Stigma and smoking. The consequences of our good intentions. Soc Serv Rev.

[CR23] Villano JL, Nee J, Coker AL, Goswami V, Hatfield ML (2015) Suicide in cancer patients: an epidemiology study. J Clin Oncol 33(15)-suppl. http://www.meeting.ascopubs.org/cgi/content/abstract/33/15_suppl/e20582

[CR24] Wille MM, Dirksen A, Ashraf H, Saghir Z, Bach KS, Pedersen JH (2016). Results of the randomized Danish lung cancer screening trial with focus on high-risk profiling. Am J Respir Crit Care Med.

[CR20] Yang Q, Huaxia L, Chunling Y, Shuyu J, Lei L (2014). Reliability and validity of Chinese version of Cataldo Lung Cancer Stigma Scale. Int J Nurs Sci.

